# The contribution of *β*-amyloid, Tau and *α*-synuclein to blood–brain barrier damage in neurodegenerative disorders

**DOI:** 10.1007/s00401-024-02696-z

**Published:** 2024-02-12

**Authors:** Ying-Chieh Wu, Tizibt Ashine Bogale, Jari Koistinaho, Marina Pizzi, Taisia Rolova, Arianna Bellucci

**Affiliations:** 1 Neuroscience Center, HiLIFE, University of Helsinki, Helsinki, Finland; 2 Department of Molecular and Translational Medicine, University of Brescia, Viale Europa 11, 25123 Brescia, BS Italy; 3 Department of Acute Brain and Cardiovascular Injury, Istituto Di Ricerche Farmacologiche Mario Negri IRCCS, Milan, Italy

**Keywords:** Amyloid *β*, Tau, *α*-synuclein, Brain macrophages, Pericytes, Endothelial cells

## Abstract

Central nervous system (CNS) accumulation of fibrillary deposits made of Amyloid *β* (A*β*), hyperphosphorylated Tau or *α*-synuclein (*α*-syn), present either alone or in the form of mixed pathology, characterizes the most common neurodegenerative diseases (NDDs) as well as the aging brain. Compelling evidence supports that acute neurological disorders, such as traumatic brain injury (TBI) and stroke, are also accompanied by increased deposition of toxic A*β*, Tau and *α*-syn species. While the contribution of these pathological proteins to neurodegeneration has been experimentally ascertained, the cellular and molecular mechanisms driving A*β*, Tau and *α*-syn-related brain damage remain to be fully clarified. In the last few years, studies have shown that A*β*, Tau and *α*-syn may contribute to neurodegeneration also by inducing and/or promoting blood–brain barrier (BBB) disruption. These pathological proteins can affect BBB integrity either directly by affecting key BBB components such as pericytes and endothelial cells (ECs) or indirectly, by promoting brain macrophages activation and dysfunction. Here, we summarize and critically discuss key findings showing how A*β*, Tau and *α*-syn can contribute to BBB damage in most common NDDs, TBI and stroke. We also highlight the need for a deeper characterization of the role of these pathological proteins in the activation and dysfunction of brain macrophages, pericytes and ECs to improve diagnosis and treatment of acute and chronic neurological disorders.

## Introduction

Amyloid *β* (A*β*), Tau and *α*-synuclein (*α*-syn) are amyloidogenic proteins forming insoluble fibrillary deposits with *β*-sheet structure in the brain of patients suffering from major neurodegenerative disorders (NDDs). Alzheimer's disease (AD), the most common among NDDs, is characterized by brain accumulation of extracellular A*β* aggregates known as plaques and intraneuronal Tau deposits called neurofibrillary tangles, that are also found in several other NDDs, including frontotemporal dementia, Pick's disease, corticobasal degeneration, progressive supranuclear palsy, argyrophilic grain disease as well as chronic traumatic encephalopathy, a condition where neuronal loss results from repetitive blast or concussive injuries [125]. Intraneuronal and intraneuritic *α*-syn aggregates, referred to as Lewy bodies (LB) and Lewy neurites, are distinctive features of Parkinson's disease (PD) and LB dementia [50]. Moreover, *α*-syn fibrillary aggregates also accumulate in glial cytoplasmic inclusions, which are typically observed in oligodendrocyte cells in the brain of patients affected by multiple system atrophy.

Aging, genetic variants and/or environmental stressors are major risk factors for the deposition of A*β*, hyperphosphorylated Tau and *α*-syn aggregates, and consequently, for the onset of the associated NDDs. Moreover, acute brain injuries, such as traumatic brain injury (TBI) or ischemia, can also trigger the accumulation of these pathological proteins in the brain, potentially leading to the development of neurodegeneration [17, 25, 30, 74, 147].

Although the area affected by different proteinopathies, as well as the sites of neurodegeneration, can markedly vary among NDDs, TBI and stroke, it is worth considering that the coexistence of A*β*, Tau, and *α*-syn pathologies has been frequently observed in these conditions, including the LB variant of AD or the recurrent presence of tauopathy in PD and LB dementia. This suggests the possibility of a synergistic and toxic interplay of A*β*, Tau and *α*-syn in promoting central nervous system (CNS) damage [120].

Compelling evidence supports that the deposition of A*β*, hyperphosphorylated Tau and *α*-syn not only participates in neuronal damage but also in blood-brain barrier (BBB) disruption, that is another main common manifestation of NDDs, TBI and stroke [11, 34, 37, 55, 130].

The BBB is a complex and finely regulated interface that protects CNS neurons by limiting the trafficking of blood components to the brain [45, 75]. In acute and chronic NDDs, it becomes more permeable to solutes, and allows an increase in lymphocyte trafficking and brain infiltration of innate immune cells [75]. Multiple rodent studies have shown that A*β*, Tau and *α*-syn pathology can disrupt brain vascular homeostasis either by directly interacting with BBB cell components or by promoting a neuroinflammatory state, that can severely perturb BBB permeability [11, 33, 34, 37, 77, 132]. The resulting BBB disruption can in turn contribute to brain damage by affecting brain homeostatic responses. As a representative example, cerebral amyloid angiopathy (CAA) pathology, characterized by A*β* accumulation within cerebral blood vessels, has been correlated with cerebral atrophy and advancing cognitive deterioration and can also precipitate hemorrhagic stroke.

In this review, we summarize and discuss growing evidence supporting the hypothesis that A*β*, Tau and *α*-syn pathology disrupts BBB integrity in acute and chronic neurological disorders through three key cellular subtypes: brain macrophages, pericytes and endothelial cells (ECs) with the aim to open insightful perspectives for the identification of innovative disease biomarkers or therapeutic targets for these disabling conditions. Although astrocytes play a critical role in the maintenance of BBB integrity, they are outside the scope of this review.

## Organization of the BBB

**Fig. 1:**
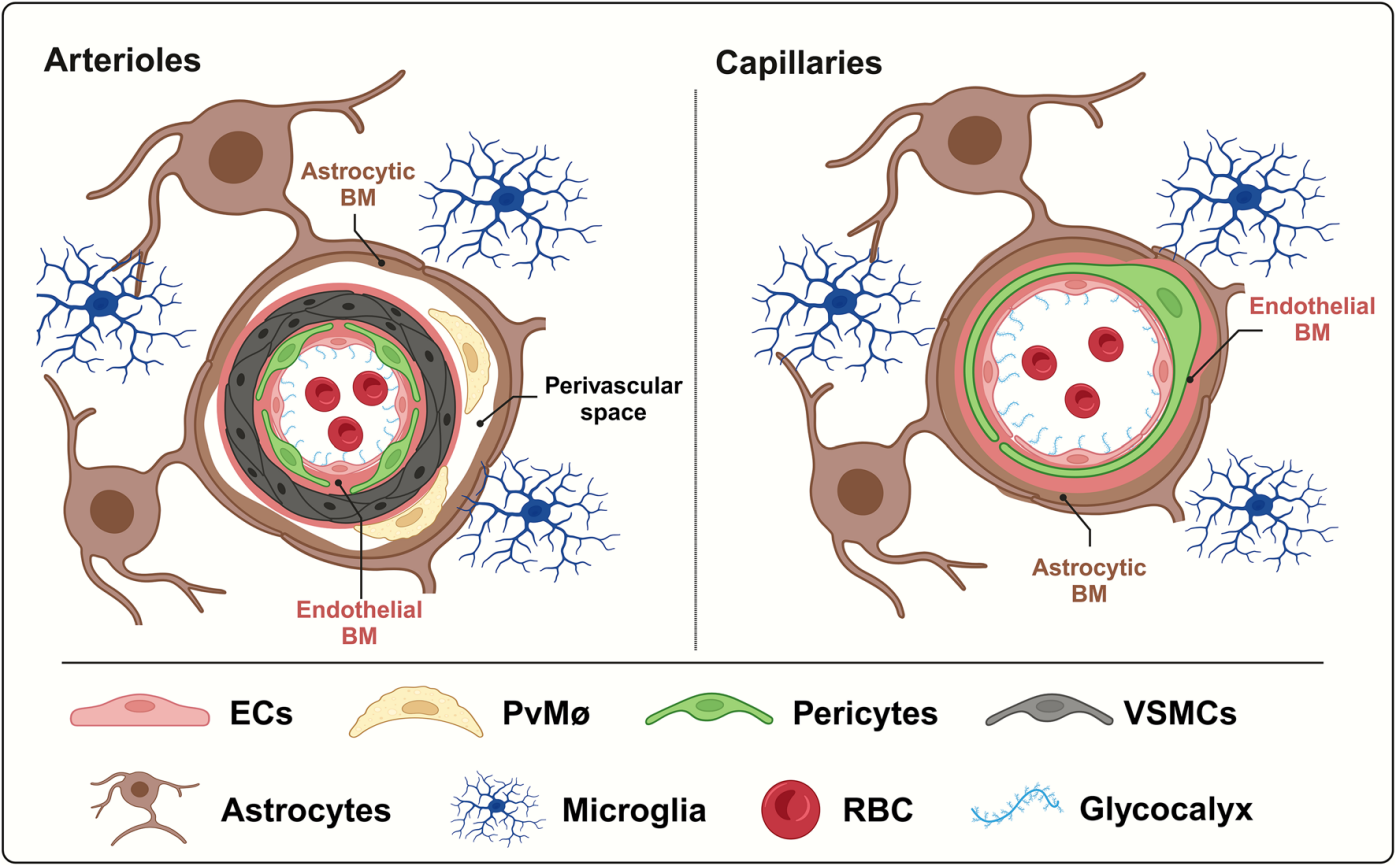
Schematic representation of the different cellular components of the BBB at arterioles and capillaries

The BBB represents the main interface between the brain and the external environment, as it restricts the transportation of molecules and regulates the trafficking of immune cells between circulation and brain parenchyma. It is a specialized configuration of the cerebral vasculature, comprising ECs enclosed by the endothelial basement membrane, where pericytes, whose extensive cytoplasmic processes wrap around ECs, are embedded (Fig. 1). Astrocyte endfeet, which also secrete their own basement membrane, constitute the most external layer of the BBB [45] (Fig. 1). In arteries and arterioles, the pericytes embedded in the endothelial basement membrane are surrounded by the vascular smooth muscle cells (VSMCs), that are distanced from astrocyte endfeet by the perivascular space, also known as Wirchow-Robin space, where perivascular macrophages (PvMΦ) reside (Fig. 1). A similar BBB organization can be found in veins, where VSMCs are organized in a thinner layer. In capillaries, the pericytes, embedded in the endothelial basement membrane closely interfaced with glia limitans, leave no perivascular space [122] (Fig. 1). PvMΦ, together with meningeal and choroid plexus macrophages, are commonly referred to as CNS (or border)-associated macrophages (CAMs) and can participate in maintaining BBB integrity by scavenging harmful molecules deriving from the bloodstream, cerebrospinal fluid (CSF) or CNS and promoting efficient immune surveillance [47].

Altogether, the above-cited BBB components participate in protecting the CNS and in orchestrating the physiological functions of the BBB, including the regulation of cerebral blood flow (CBF) (Fig. 1).

Cerebrovascular ECs have distinctive characteristics when compared to their counterparts outside the brain as they are strictly joined by tight junctions, express low levels of leukocyte adhesion molecules, exhibit low rates of micropinocytosis and caveolar transcytosis, and possess a plethora of substrate-specific transport systems. These mechanisms regulate the influx of essential nutrients into the brain or the efflux of unwanted substances into the bloodstream [45, 71]. Moreover, these cells secrete a peculiar glycocalyx consisting of a closely knitted network of glycosaminoglycans that coat the luminal surface of blood vessels ensuring elevated luminal surface coverage and low permeability [45].

The endothelial and astrocyte basement membranes, crucial in determining BBB permeability to leukocytes, are composed of an extracellular highly organized amorphous matrix of structural proteins including collagen IV family proteins, nidogens, heparan sulfate proteoglycans and laminins [52]

Pericytes, originating from both the neural crest and mesoderm during development [40, 78], reside in the endothelial basement membrane within microvessel walls, including capillaries, veins, venules and arterioles. Studies indicated that 40–80% of brain ECs are covered by pericytes, varying with brain region, species, and analysis method [51, 107]. Pericytes are believed to play an important role in BBB maintenance, the regulation of capillary diameter, and angiogenesis [19]. VSMCs, situated in large-diameter vessels such as arteries and veins, serve similar functions to pericytes in stabilizing vasculature morphology and functions. Pericytes and ECs co-produce the basement membrane and establish adherens and gap junctions between each other [133]. Adherens junctions connect the cytoskeletons of the two cell types, mediating contact inhibition through contractile forces. Meanwhile, gap junctions interconnect their cytoplasms, facilitating the passage of metabolites and ionic currents.

Astrocyte endfeet interdigitates and overlaps, providing nearly complete coverage of the BBB and forming its outermost layer. Additionally, astrocytes produce substances that affect BBB integrity such as angiotensin II, angiopoietin-1 or sonic hedgehog, that in turn affect ECs homeostasis [140, 142].

Finally, it is worth mentioning that microglial cells, the most widespread brain macrophages derived from haematopoietic precursors that migrate from the yolk sac into the CNS parenchyma [49], serve as the brain’s primary line of defence past the BBB. They play a crucial role in innate immune responses within the CNS. Juxtavascular microglia also interact with vascular areas lacking astrocyte endfeet and can control vascular architecture and BBB permeability in both health and disease [93].

## ECs, pericytes and macrophages in the maintenance of BBB integrity and regulation of CBF

Brain ECs are primarily involved in the development and maintenance of the BBB and interact with other cells of the so-called neurovascular unit, a specialized cluster of cellular and extracellular components including neurons, astrocytes, ECs, VSMCs, pericytes and extracellular matrix. The neurovascular unit detects the needs of neuronal supply and triggers necessary responses such as vasodilation or vasoconstriction, to regulate CBF and BBB function [96]. In particular, the interplay between ECs, pericytes, PvM*Φ* and microglia has a crucial role in regulating the BBB in both healthy and diseased brain [117].

Pericytes are critical for proper vascular development and stabilization by tightly wrapping around brain ECs and engaging in close functional interactions with them [19]. The depletion of pericytes, whether during development or in adulthood, has been observed to increase vascular permeability and disrupt barrier function at least partially by decreasing the expression of tight junction proteins (TJPs) and promoting leukocyte adhesion to ECs, thus highlighting the importance of pericytes in maintaining an intact BBB [4, 26]. Indeed, co-culturing ECs with pericytes in laboratory settings has been shown to enhance the expression of TJPs [26]. Additionally, pericyte contractility influences ECs sprouting and cell cycle progression, indicating their role in controlling endothelial proliferation and angiogenesis [36].

On the other hand, pericytes, like VSMCs, respond to vasoconstrictor and vasodilator signals, enabling them to contract and relax accordingly [51]. This dynamic behavior allows pericytes to control the diameter of capillaries although their role in CBF regulation has not been extensively corroborated. In summary, pericytes play a multifaceted role in vascular development, the maintenance of the BBB and the regulation of vascular functions through their interactions with ECs.

Brain macrophages are essential sentinels in the immune response as they become activated under brain injury or immunological stimuli [47] and the resulting neuroinflammation can contribute to neurodegeneration both directly as well as by impairing the BBB [56, 102, 118, 135]. Discerning the different roles of PvM*Φ* and vascular-associated microglia in BBB function has been challenging as they share cellular markers, such as Iba1 and CX3CR1, and produce similar inflammatory mediators. However, CAMs can be distinguished from microglia by their location in brain leptomeningeal and perivascular space and expression of the mannose receptor CD206, CD163 and Lyve1 [35]. Though the injection of clodronate liposomes in the ventricles and cisterna magna was reported to selectively deplete PvM*Φ* and meningeal macrophages, respectively [35, 106], technically it is difficult to achieve without affecting to some extent also parenchymal microglia or the other CAMs. More recently, the combination of single-cell RNA sequencing, time-of-flight mass cytometry and single-cell spatial transcriptomics with fate mapping and advanced immunohistochemistry has opened the possibility to identify distinctive transcriptomic profiles of CAMs [115].

It has long been known that cytokines and reactive oxygen species (ROS) produced by macrophages and microglia during inflammatory conditions can induce BBB damage and immune cell infiltration. Vessel-associated microglia, CAMs and ECs act in concert to regulate BBB tightness, angiogenesis and CBF in health and disease [27]. For instance, in the steady-state conditions PvM*Φ* can promote BBB integrity in vitro and in vivo [57]. Depletion of microglia/macrophages in the striatum decreases the integrity of blood vessels and increases the levels of inflammatory cytokines [53]. Both macrophages and parenchymal microglia have also been shown to promote angiogenesis, especially in tumor conditions [14]. Perivascular microglia play a detrimental role following stroke either indirectly, through the production of pro-inflammatory cytokines and chemokines which in turn promote ECs activation, or directly by interacting with ECs and engulfing them, thus leading to vessel disintegration [69]. In contrast to macrophages, juxtavascular microglia have been reported to produce Claudin 5, a member of TJPs, to physically interact with ECs and promote vessel injury repair during systemic inflammation [56]. However, during sustained inflammation, activated microglia phagocytose astrocytic endfeet, thus contributing to BBB damage [56].

Much remains unknown regarding signalling pathways involved in the interaction between cerebral ECs and microglia/macrophages in various neurological disorders. Given the anatomical proximity of PvM*Φ* to ECs of brain vessels and the increasing evidence for the role of these cells in neurodegenerative diseases, it is worth speculating that they play an even superior role in BBB integrity. Therefore, more future studies on the interplay between PvM*Φ* and ECs in the brain are warranted.

Finally, a reciprocal modulatory interaction between pericytes and microglia has also been highlighted [94]. For instance, microglia can control pericyte maturation, number and apoptosis [48, 141]. On the other hand, pericytes can control microglial function, homeostasis and motility by releasing cytokines, such as interleukin 6, and chemokines including C-X3-C Motif Chemokine Ligand 1, Monocyte chemoattractant protein-1, interleukin 8, and C–C Motif Chemokine Ligand 5 [124].

These findings support that the interplay between ECs, pericytes and microglia/macrophages plays a crucial role in maintaining BBB homeostasis and integrity and suggests that insults impacting these different cell populations, including the acute and progressive accumulation of pathological proteins, can perturb their reciprocal inter-regulatory function resulting in brain vascular damage.

## The contribution of A*β*, Tau and *α*-syn to BBB damage in NDDs

Numerous studies have emphasized the effect of A*β*, Tau and *α*-syn pathology on BBB integrity. In particular, evidence from experimental models has highlighted that these pathological proteins not only cause BBB damage through the activation of brain macrophages that in turn promote neuroinflammation but also affect ECs and pericyte function. Below, we summarize the main findings describing the effect of A*β*, Tau and *α*-syn on the BBB through the modulation of the interplay between brain macrophages, ECs and pericytes.

### A*β*, Tau and *α*-syn pathology as damage-associated molecular patterns (DAMPs) triggering brain macrophage activation

Since in the brain of patients affected by NDDs A*β*, Tau and *α*-syn are continuously generated, failure to remove them results in chronic neuroinflammation that, in addition to contributing to neuronal damage, further increases BBB permeability and vascular dysfunction [54, 102, 148]. Amyloidogenic proteins can initiate a sterile immune response by acting as DAMPs that activate pattern recognition receptors, such as toll-like receptors (TLRs), on cerebral myeloid cells [54, 148].

Soluble parenchymal A*β* (and other amyloids) can drain into the perivascular space [63], where it encounters PvM*Φ*. Macrophages, as the resident immune cells within the vasculature, are professional phagocytes and can efficiently engulf amyloid aggregates. High amyloid load and cellular stress can impair phagocytosis function, leading to macrophage cell death and exacerbating CAA pathology [143]. Moreover, a recent study has shown that PvM*Φ* regulates the flow rate of CSF, which decreases with aging, further reducing the rate of amyloid clearance [35].

The degree of the pro-inflammatory factor release depends on the receptors involved. Receptors like scavenger receptor (SR)-A, SR-BI and triggering receptor expressed on myeloid cells 2 promote the uptake of extracellular amyloid deposits without eliciting a significant inflammatory response, whereas TLR2/4 and CD36 stimulation induces the production of neurotoxic cytokines and ROS via nuclear factor κB (NFκB) and NLRP3-ASC-inflammasome pathways, thus promoting neuronal and vascular degeneration [54, 64, 131]. Reduction in SR-A and SR-BI impairs PvM*Φ* function and enhances CAA [83, 131], while CD36 deficiency in PvM*Φ* is protective in transgenic mouse models [135].

In addition, post-mortem and experimental studies have shown that neurons with Tau pathology are surrounded by reactive microglia/macrophages [8, 9], suggesting that intraneuronal pathological Tau also activates brain macrophages. Indeed, neurons with Tau filaments expose abnormally high levels of phosphatidylserine triggering opsonin milk-fat-globule EGF-factor-8-mediated phagocytosis by microglia, that in turn become hypofunctional [15, 16]. Phagocytosed Tau has also been reported to trigger inflammatory activation via polyglutamine binding protein 1 cyclic GMP-AMP synthase-Stimulator of interferon genes-NFκB pathway [66].

Other evidence supports that the NLRP3 inflammasome pathway can be induced by fibrillary *α*-syn or by *α*-syn-pathology-dependent dopaminergic failure, which results in the reduction of microglia dopamine D1 and D2 receptor stimulation and consequent microglia activation [105, 126].

By compromising BBB permeability and increasing the expression and binding affinity of intercellular adhesion molecule 1 and vascular cell adhesion molecule 1 on ECs, pathological protein-related neuroinflammatory cascades also contribute to the recruitment, activation and extravasation into the parenchyma of peripheral immune cells, including neutrophils and T cells, into the brain [104, 144, 145]. In turn, the accumulation of peripheral leukocytes in the blood vessels and perivascular space can decrease blood circulation and increase BBB leakage, thus further contributing to brain damage [10, 24].

In support of the key role of neuroinflammation in T cell recruitment there is evidence indicating that the brain areas exhibiting marked neuroinflammation and microglial activation in post-mortem PD brains frequently show the presence of leukocytes next to MHCII-positive astrocytes in proximity to blood vessels [61, 111]. Another study described that CAMs are localized in close proximity to T cells in post-mortem PD brains and showed that these cells act as the main antigen-presenting cells necessary to initiate a CD4 T cell recruitment and neuroinflammation in response to *α*-syn accumulation [118]. Similar findings have been described in post-mortem brains of AD and LB dementia patients, where significant T lymphocyte recruitment to both grey and white matter has been reported [3, 134].

Collectively, these observations support that A*β*, Tau and *α*-syn pathology act as main DAMPs for brain-macrophage activation-related BBB disruption.

### Impact of A*β*, Tau and *α*-syn pathology on ECs

The exact origin of A*β* depositions in the vasculature is not fully understood yet, but evidence suggests that neurons and astrocytes are the main sources, with subsequent spreading or movement towards the vasculature for clearance [39]. However, recent findings also support that ECs may contribute to the production of A*β* and CAA [128].

A*β* exerts both direct or indirect effects on ECs within brain microvasculature by altering the distribution of TJPs, promoting ECs death, elevating oxidative stress and inducing proinflammatory cytokine production in glial cells [130, 145]. A*β* accumulation adversely affects brain vessel walls, leading to BBB damage and potential hemorrhage in CAA mouse models and human patients [42, 55]. In vitro studies have shown that A*β* exposure disrupts actin organization and induces apoptosis in ECs [129]. Furthermore, CAA patients and amyloid precursor protein (APP)-overexpressing mice exhibit decreased TJPs expression and increased levels of matrix metalloproteinases (MMPs) [55]. The presence of A*β* also hampers endothelial nitric oxide synthase/heat shock protein 90 interaction [80] and promotes the formation of von Willebrand factor (VWF) fibers, which contribute to inflammatory and thrombogenic responses in brain vessels [121]. These findings highlight the detrimental effects of A*β* deposition on vessel walls, including ECs impairment, compromised BBB and alterations in inflammatory and thrombogenic responses. All these mechanisms can contribute to the impairment of neurovascular control.

The accumulation of Tau oligomers in cerebral microvessels has been reported in human AD, LB dementia and progressive supranuclear palsy patients [22]. Microbleeds have been detected in the brains of patients affected by frontotemporal dementia [28] and cerebrovascular inflammation has been associated with Tau pathology in Guam parkinsonism dementia and chronic bilirubin encephalopathy. In these cases, areas with significant accumulation of neurofibrillary tangles exhibited upregulation of adhesion molecules, disruption of TJPs, morphological alterations in brain microvessels, including thickening of the vessel wall, vessel lumen reduction as well as increased in collagen-type IV content *per* vessel [84]. Still, Tau pathology is associated with small vessel disease [70] and immune cells trafficking across the BBB also appears to be modulated by neurofibrillary pathology in Tauopathies [85]. In P301S transgenic mice, brain microvascular ECs uptake soluble pathogenic Tau oligomers from the extracellular space, which over time accumulate within ECs further leading to mitochondrial damage and ECs senescence [60]. Aged Tau-overexpressing mice were found to develop cortical blood vessel changes such as abnormal spiralling morphologies and reduced diameters in parallel to increased vessel density [10]. These changes were accompanied by alteration in cortical CBF and increased expression of angiogenesis-related genes such as *Vegfa*, *Serpine1*, and *Plau* in CD31-positive ECs [10], hinting that Tau pathological changes in neurons can impact brain ECs biology, thus altering the integrity of brain microvasculature. This observation is corroborated by the fact that other lines of Tau transgenic mice exhibit progressive BBB leakage with IgG, T cell, and red blood cell infiltration that is prevented by Tau depletion [11].

Numerous studies support that *α*-syn can differentially impact on ECs homeostasis as previously reviewed [13]. For instance, transgenic expression of the human A53T mutated form of the protein in mouse brains decreases the expression of TJPs resulting in increased vascular permeability and also leads to the accumulation of oligomeric *α*-syn in activated astrocytes that release vascular endothelial growth factor (VEGF) A and nitric oxide [81], key regulators of ECs homeostasis. Moreover, toxic forms of *α*-syn can modulate ECs function by limiting the release of inflammatory cytokines and adhesion molecules from their secretory granules or downregulating the expression of TJPs [72, 79]. This has been recently confirmed in an innovative human brain-on-a-chip model of the substantia nigra and containing dopaminergic neurons, astrocytes, microglia, pericytes, and microvascular brain ECs cultured under fluid flow and exposed to synthetic *α*-syn preformed fibrils [103]. By using this model, authors found that *α*-syn synthetic fibrils impair BBB permeability by directly interacting with ECs and altering the expression of vascular channels, TJPs and other key genes involved in BBB homeostasis, including extracellular proteases of the Serpin family and collagens.

### Impact of A*β*, Tau and *α*-syn pathology on pericytes

Pericyte degeneration is associated with A*β* deposition [137], facilitating the progression of neurodegenerative pathology [114]. Consistently, a correlation between A*β* accumulation and pericyte loss in patients with NDDs and transgenic mouse models has been demonstrated [91, 114]. Interestingly, though A*β*42 is commonly considered more cytotoxic than A*β*40 due to its aggregation-prone nature, A*β*40 has been found to exert similar effects on pericytes. In patients with AD, a decreased population of NG2-positive pericytes in the hippocampus was observed, negatively correlating with A*β*40 levels. In vitro experiments revealed that exposure to A*β*40 monomers enhanced pericyte viability and proliferation while reducing caspase 3/7 activity. However, exposure to fibril-enriched A*β*40 resulted in decreased pericyte viability and proliferation, along with increased caspase 3/7 activity [119]. These findings suggest that A*β*40 may serve as a key regulator of the pericyte population in the brain, both in disease and in healthy conditions. The loss of pericytes reduces TJPs expression in ECs, compromising BBB integrity and increasing A*β* load in the brain [107].

Similarly, a study on a human-induced pluripotent stem cell-derived BBB model comprising astrocytes, ECs and pericytes suggested that pericytes play a key role in the intravascular accumulation of A*β* [12].

Moreover, A*β* has been discovered to induce pericyte-mediated constriction of brain capillaries through the upregulation of endothelin-1 release, which is mediated by the overproduction of ROS [98]. In vitro studies further support that exposure to A*β* oligomers leads to the development of a hypercontractile phenotype in pericytes [58]. Increased constriction of brain microvessels by hypercontractile pericytes likely contributes to the reduction in CBF observed in NDDs.

Other evidence indicates that following repeated head trauma in mice both types of mural cells, pericytes and VSMCs, uptake recombinant human tau more efficiently as compared to astrocytes and ECs [100]. Interestingly, other studies support that VSMCs undergo significant phenotypic changes and acquire an inflammatory phenotype under AD-like conditions, coinciding with Tau hyperphosphorylation [2]. Although evidence regarding Tau pathology in pericytes is limited, it can be hypothesised that this pathological protein may similarly impact both mural cell types.

Finally, recent studies have shed light on the significant involvement of pericytes in *α*-syn pathology. In vitro investigations have demonstrated that pericytes can contribute to the clearance of *α*-syn aggregates by internalizing and degrading them [32, 127]. However, under conditions of additional cellular stress, this process can lead to increased production of ROS in pericytes, ultimately resulting in their apoptosis [127]. Other cell culture studies showed that monomeric *α*-syn can lead to BBB dysfunction through activated brain pericytes releasing inflammatory mediators such as interleukin 1*β*, interleukin 6, tumor necrosis factor-*α*, monocyte chemotactic protein-1 and MMP-9 [34]. Additionally, studies utilizing primary brain pericytes obtained from individuals with PD have shown the formation of tunneling nanotubes, which are membranous channels composed of F-actin and facilitate the transfer and spread of *α*-syn between cells [31]. These findings highlight the complex involvement of pericytes in *α*-syn pathology, which appears to encompass both beneficial and detrimental effects.

## The contribution of A*β*, Tau and *α*-syn to BBB damage in stroke

Stroke patients have a high risk of developing cognitive decline or parkinsonism, suggesting that pathological protein accumulation induced by brain ischemia can significantly contribute to neurodegeneration. Clinical studies highlighted a significant risk of developing dementia symptoms and progressive cognitive decline after ischemic and hemorrhagic strokes [89]. Post-stroke dementia commonly manifests in patients, particularly as they age, and post-mortem analysis of stroke patients' brain samples has revealed the presence of A*β* deposition [65]. Animal models have also shown increased A*β* deposition and CAA pathology following stroke, suggesting a potential role of A*β* in post-stroke dementia development [46]. One proposed mechanism is the disruption and dysfunction of the BBB after stroke, impairing the clearance of A*β* from the brain to the circulation [46]. Chronic cerebral hypoperfusion dysregulates the expression of low-density lipoprotein receptor-related protein 1 and receptor for advanced glycation endproducts, key mediators of A*β* transport across the BBB and out of the brain, thus leading to A*β* accumulation in the vessel walls and parenchyma [5]. Additionally, the glymphatic system, the brain’s waste disposal system [95] which can also mediate A*β* clearance, may also be affected after stroke, contributing to A*β* buildup [44]. However, a recent review integrating data from clinical and pre-clinical studies has revealed inconsistencies in the relationship between post-stroke A*β* deposition and cognitive impairment, observed in both human patients and rodent models [101].

Conversely, A*β* accumulation itself can increase the risk and severity of stroke [59, 110]. Deposits of A*β* in the vasculature can lead to VSMCs loss and destabilize vessel wall, elevating the risk of hemorrhagic stroke [138]. CAA pathology exacerbated ischemic damage in a mouse model [90]. More evidence is necessary to gain a better understanding of the relationship between A*β* accumulation and stroke, as well as how A*β* may influence the progression of both conditions.

Recent findings support that *α*-syn and hyperphosphorylated Tau accumulation mediates and promotes stroke-induced brain damage and possibly contributes to post-stroke cognitive impairment [73, 97, 116, 136]. Positron emission tomography/magnetic resonance imaging studies have indeed shown that neurofibrillary tangles can form after ischemic stroke and spread in the peri-ischemic brain parenchyma, while total Tau levels in the CSF positively associate with measures of brain atrophy one-year post-stroke [62]. The levels of oligomeric and Ser129-phosphorylated *α*-syn, which are considered possible pathological biomarkers for PD, are increased in red blood cells from acute ischemic stroke patients [139]. Furthermore, it has been described that *α*-syn accumulation promotes glycogen synthase kinase (GSK)-3*β*-mediated phosphorylation of Tau following transient focal ischemia in mice [87].

Reinforcing the relevance of Tau and *α*-syn in stroke, it has been observed that Tau and *α*-syn knockout mice exhibit significantly smaller brain damage after transient focal ischemia when compared to wild-type mice [73, 82, 87]. Moreover, reducing Tau hyperphosphorylation with GSK-3*β* inhibitors such as nimodipine or by the administration of glucosamine, which by promoting O-GlcNAcylation antagonizes phosphorylation, has shown neuroprotective effects in rodent models of ischemia [20, 87]. Additionally, task-specific training rehabilitation and an enriched environment improved functional deficits and reduced Tau-phosphorylation and neuroinflammation in the phototrombotic ischemia rat model [67], suggesting that brain plasticity mechanisms may modulate Tau pathology following brain ischemia. Finally, it has been described that chronic cerebral hypoperfusion in a rat model of post-stroke dementia aggravated cognitive impairment and Tau hyperphosphorylation by interfering with Tau clearance through the glymphatic system [6]. This aligns with previous findings showing that chronic cerebral hypoperfusion can enhance Tau hyperphosphorylation and impair autophagy in an AD mouse model [108], suggesting that ischemia may perturb the mechanisms of pathological Tau elimination.

Hence, though the exact contribution of Tau and *α*-syn accumulation to post-ischemic vascular damage remains to be determined, these findings indicate that *α*-syn accumulation and Tau hyperphosphorylation significantly contribute to post-stroke secondary brain damage and may be possibly considered novel therapeutic targets for stroke therapy. It can be inferred that given the significant role of these proteins in the modulation of microglia/macrophage and BBB cell activation, their accumulation can significantly impact vascular injury thus warranting further studies on this topic.

## The contribution of A*β*, Tau and *α*-syn to BBB damage in TBI

TBI has immediate and devastating effects, often triggering long-term neurodegeneration involving proteins like A*β*, Tau, and *α*-syn. Studies support that a history of TBI is a risk factor for AD in males [43]. The pathological connection between TBI and AD is supported by the observation of increased A*β* plaques and soluble A*β* species in brain tissue following TBI [29]. Similar acute A*β* accumulations accompanied by neuronal death and memory impairment have been reported in APP-transgenic mice subjected to TBI [123].

Moreover, traumatic axonal injury, a common pathology seen after TBI, provides a potential mechanism for A*β* production. The prevailing hypothesis suggests that the abundant APP, which accumulates in damaged axons, undergoes aberrant cleavage to form A*β*, subsequently aggregating as A*β* plaques [68]. The accumulation of A*β* not only leads to axonal degeneration but also induces neuronal inflammation and neuronal death, thereby contributing to the progression of long-term neurodegenerative diseases.

Several research reports showed the occurrence of pathological hyperphosphorylated Tau accumulation and spreading following TBI, while the ratio between phosphorylated and total Tau in plasma serves as a peripheral biomarker of acute and chronic TBI [112]. In particular, it has been proposed that neuroinflammation and Tau pathology mutually participate in inducing cognitive decline following TBI [23]. Tau/A*β*-induced BBB damage is also believed to initiate a deleterious feed-forward loop contributing to TBI-associated vascular damage [109]. Indeed, markers of vascular injury have been associated with hyperphosphorylated Tau pathology in chronic traumatic encephalopathy [74]. Furthermore, post-mortem studies on the brain of a former professional boxer diagnosed with chronic traumatic encephalopathy and comorbid schizophrenia showed that areas with extensive accumulation of hyperphosphorylated Tau protein exhibited BBB damage with a decrease in Claudin 5 and enhanced extravasation of endogenous blood components such as fibrinogen and IgG [41]. This finding aligns with previous studies on patients affected by dementia pugilistica, which showed microvascular damage in association with Tau pathology [86].

It has been reported that *α*-syn is also increased in the post-mortem brain of subjects with TBI [1, 21] as well as in the CSF of TBI patients [92]. This increase in *α*-syn following TBI might explain the elevated risk of subsequently developing PD [1, 18] and has been corroborated by animal studies [21]. Evidence showing that lentivirus-mediated downregulation of *α*-syn reduces neuroinflammation and promotes functional recovery in rats with spinal cord injury [146] also supports that *α*-syn can contribute to inflammation-associated vascular damage in TBI.

## Conclusions and authors’ perspectives

**Fig. 2:**
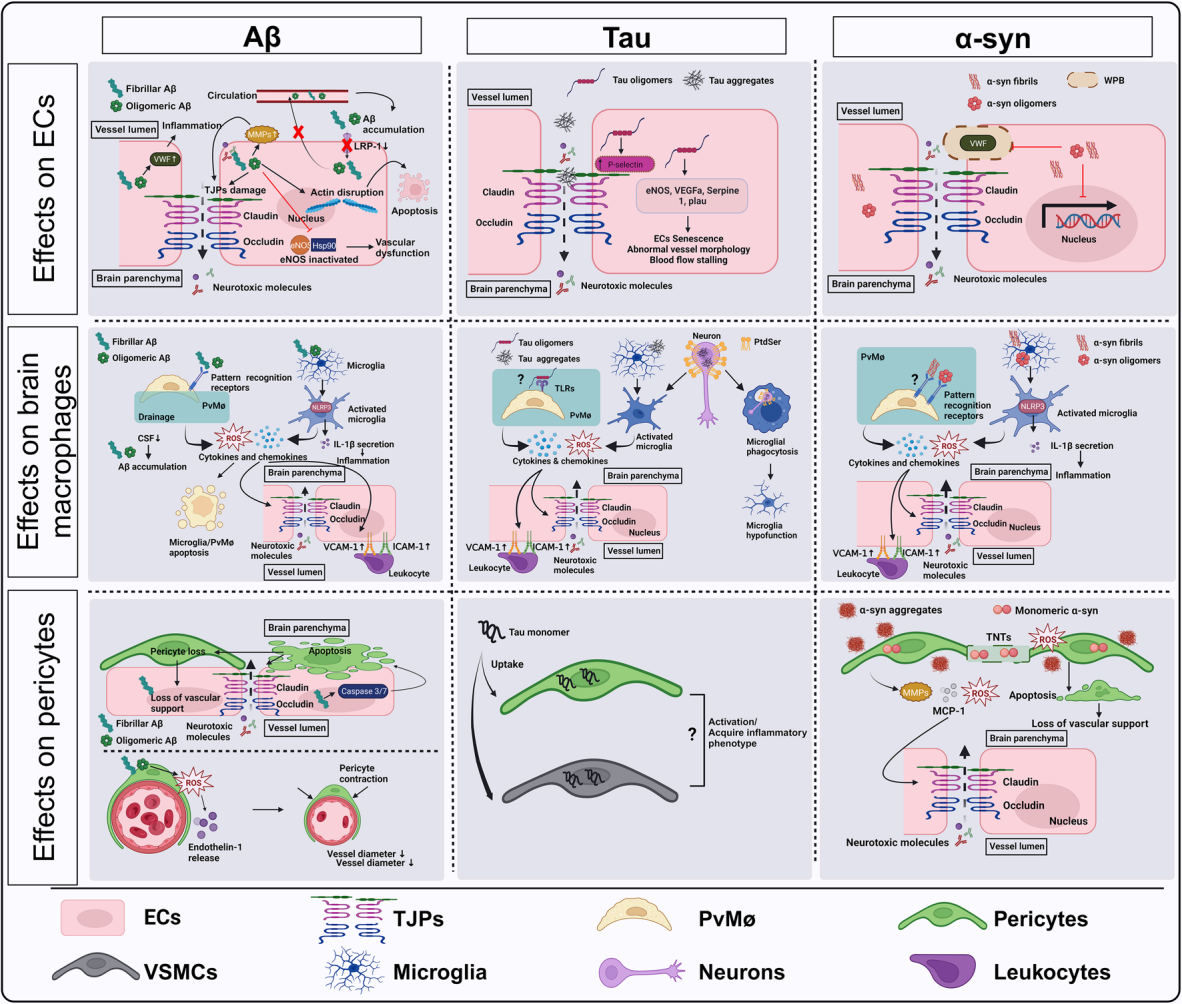
Overview of the impact of A*β*, Tau and *α*-syn on ECs, brain macrophages and pericytes. A*β* oligomers and fibrils can enter in ECs producing their activation and damaging TJPs eventually resulting in ECs death. While extracellular Tau fibrillary aggregates can damage TJPs, Tau oligomers can also enter ECs and induce their activation that in turn can produce ECs senescence, thus altering vessel morphology and causing blood flow stalling. ECs can uptake *α*-syn fibrils and oligomers that can interfere with ECs function, including VWF release. Extracellular A*β*, Tau and *α*-syn fibrillary aggregates and oligomers have been found to activate brain macrophages mainly by acting as DAMPs. Intracellular Tau aggregates can also produce a peculiar neuronal pro-inflammatory phenotype with Phosphatidylserine (PtdSer) membrane exposure that stimulates their phagocytosis by microglial cells, which consequently become hypofunctional. Brain macrophage activation induced by A*β*, Tau, and *α*-syn leads to oxidative stress, generating reactive oxygen species (ROS) and producing pro-inflammatory cytokines. This cascade of events subsequently impacts ECs, contributing to compromised BBB integrity. Additionally, pro-inflammatory cytokines derived from activated brain macrophages and ECs may stimulate ECs expression of intercellular adhesion molecule 1 (ICAM-1) and vascular cell adhesion molecule 1 (VCAM-1), thus attracting leukocyte adhesion from circulation. A*β*, Tau and *α*-syn can also differentially affect pericytes. A*β* can induce pericyte degeneration with subsequent loss of vascular support. A*β* also stimulates pericyte contraction by promoting endothelin release, thus impacting CBF. Tau monomers can enter pericytes stimulating their activation and dysfunction following repeated head trauma. Pericytes can be activated by extracellular *α*-syn aggregates accumulation and by the uptake of high levels of monomeric *α*-syn, which can then be transmitted between pericytes through tunnelling nanotubes (TNTs). In turn, pericytes can become hypofunctional and there is a loss of their trophic support on ECs

Impairment of the BBB occurs in many neurological disorders, including acquired brain injury conditions such as stroke or TBI, as well as NDDs such as amyloidosis, tauopathies and synucleinopathies. In these conditions, blood vessels are usually damaged secondary to the pathological insult as a result of the activation of the intrinsic cellular mechanisms of neuroinflammation. BBB damage mainly occurs as a result of alterations in TJPs expression, the activation state of ECs, pericytes and astrocytes or impaired angiogenesis [99]. These processes can be triggered by brain inflammation or can be directly initiated by the accumulation of pathological proteins. As highlighted in this review, A*β*, Tau and *α*-syn can contribute to brain vascular damage mainly by inducing brain macrophage activation or by affecting pericytes and ECs (Fig. 2). This is further supported by findings showing a correlation between vascular risk and A*β* and Tau load in individuals with cognitive decline [76]. Additionally, conditions like CAA can lead to diffuse ischemic brain injury and predisposes individuals to hemorrhagic stroke or intracranial and subarachnoid hemorrhage [59, 110].

Given the pivotal role exerted by the BBB in the maintenance of brain homeostasis and in CNS protection, it is predicted that BBB dysfunction can more or less severely contribute to the onset and progression of brain damage in neurological disorders. Consistently, BBB disruption has been associated with severe and detrimental outcomes in the context of many neurological disorders [99].

Since BBB injury is relevant in many CNS disorders, identifying novel therapeutic targets to counteract BBB dysfunction is crucial and compelling. In this framework, we propose that targeting A*β*, Tau and *α*-syn pathology constitutes an attractive therapeutic avenue to limit BBB damage in NDDs. From the manyfold studies on NDDs we know that using A*β*, Tau and *α*-syn immunotherapy or gene-silencing therapy raises concerns about potential protein-depleting detrimental effects and the risk of further vascular damage. In general, the re-establishment of the physiological function of these proteins may be more beneficial than clearing them from the brain, as this would also imply a loss of their physiological function. Nevertheless, targeting these proteins in the context of the acute phases of stroke or TBI may be evaluated as a possible option to attenuate vascular injury. For instance, though immunization may be problematic in the context of acute brain injury, gene silencing by antisense oligonucleotide (ASO) administration may be a valuable strategy to reduce the burden of toxic forms of A*β*, Tau and *α*-syn in stroke or TBI. Since some gene silencing approaches have reached clinical trial [38] and many other are in preclinical development, their repurposing may be easily achievable in the next few years.

Moreover, the levels of pathological proteins in the peripheral fluids of patients following acute brain injury may serve as potential prognostic biomarkers for these disabling conditions. This implies that current diagnostic assays allowing the assessment of A*β*, total and phosphorylated Tau or *α*-syn in amyloidosis, Tauopathies and synucleinopathies should also be re-evaluated in this context.

On the other hand, it may be feasible that some of the innovative therapeutic approaches proposed for acute brain injury, such as pro-angiogenic therapy, that is under evaluation in the treatment of stroke [113], may be useful to limit BBB damage in the context of neurodegenerative disorders with pathological protein accumulation. On this line, it has been found that VEGF therapy, which ameliorated post-ischemic brain damage following brain ischemia in gerbils [7] can also exert neuroprotective effects in PD models [88].

Though we need to deepen our understanding of the molecular underpinnings linking pathological A*β*, Tau or *α*-syn burden to BBB dysfunction, exploring their interplay may offer novel insightful perspectives for the diagnosis and treatment of acute and chronic neurological disorders.
